# Progress in Surgery of the Liver and Gall Bladder

**Published:** 1902-07-26

**Authors:** 


### Progress in Surgery of the Liver and Gall Bladder.

(*Continued from page 277.*)

**Obstructive Jaundice.**—Robson [5] bases his observations on an experience of over 200 cases. He regards a painless onset of chronic jaundice as suspicious either of chronic catarrh dependent on cancer of the liver, or of occlusion of the hepatic or common bile ducts by growth, and if this be associated with distension of the gall bladder, and rapid loss of weight and strength, cancer of the head of the pancreas will probably be found. On the other hand, the history of an attack of pain in the upper abdomen, followed within twenty-four or thirty-six hours by jaundice, and preceded by so-called "spasms," either recently or at some remote period, is strongly suggestive of cholelithiasis. In the latter case the jaundice will probably be less intense than in the former, and intermittent fever with chills and sweats will probably either be present or have been noticed at some stage of the illness. Enlargement of the liver is much more common in obstruction due to cancer than in that from gall-stones, though it may be present in either; in cancer, however, nodules or irregular bosses may be discovered, and in gall-stones an elongation of the right lobe of the liver, which is apt to be mistaken for a distended gall-bladder, can often be felt. The presence of ascites is suggestive of malignant disease. The jaundice of gall-stones is rarely continuously the same, but increases or diminishes from time to time, whereas the jaundice of obstruction due to growth steadily increases or tends to become absolute, especially in cancer of the bile duct or the head of the pancreas. Jaundice with malignant disease runs a very short course, whereas jaundice from gall-stones or some simple cause may go on for several years. Fat in the motions and glycosuria, with very rapid wasting are suggestive of pancreatic trouble. Ague-like symptoms more frequently accompany gall-stones in the common duct than malignant disease of the ducts or of the head of the pancreas, but there are exceptions. After the abdomen is opened the discovery of adhesions in the neighbourhood of the gall-bladder, and a contracted gall-bladder are strongly suggestive of gall-stones. If no gall-stones be found, but the head of the pancreas be swollen and harder than normal, the surgeon should not too hastily condemn the patient as the subject of cancer of the head of the pancreas, for the swelling may be a chronic pancreatitis, and may be curable by cholecystotomy. Before seriously interfering with adhesions, it is desirable to raise the abdominal wall and carefully examine the liver and adjoining parts so as to be assured of the absence of secondary nodules of cancer. A rigid right rectus abdominis and tenderness one inch above and to the right of the umbilicus is as suggestive of gall-stone trouble as is McBurney's tender point of appendicitis. A hæmorrhagic condition is common in cases of chronic jaundice, especially if due to cancer of the head of the pancreas or to interstitial pancreatitis. If not excessive, this can be corrected by chloride of calcium, or possibly supra-renal extract or gelatine; but if severe, it will undoubtedly seriously add to the risks of operation. If a patient at or past middle life has heart disease or albuminuria, operation should not be lightly entertained, and such cases have, as a rule, to be content with the relief that can be given by medical treatment. Deaver [6] quotes Murchison's classification of causes of obstructive jaundice as follows:—1. By foreign bodies within the ducts, as gall-stones and parasites. 2. By inflammatory tumefaction of the duodenum or of the lining membrane of the duct. 3. By stricture or obliteration of the duct. 4. By tumours closing the orifice of the duct or growing into its interior; by pressure of the duct from without, as by tumours of the liver itself, of the stomach, pancreas, kidney, or omentum. 5. By pressure of enlarged glands in the fissure of the liver, more rarely by abdominal aneurism, a fæcal accumulation, or a pregnant uterus. 6. To these may be added lowering of the blood pressure in the liver, so that the tension in the smaller bile-duct is greater than in the blood-vessels. In this class very probably may be placed the cases resulting from mental shock or depressing emotions. Berry [7] narrates a case of carcinoma of the head of the pancreas producing obstructive jaundice.

**Omentofixation for Hepatic Cirrhosis.** — Mongour's [8] statistics show this operation to be a grave one, for the mortality amounts to 35 per cent. One of the five cases reported as cured simply had ascites of cardiac origin, while in another case it is very doubtful whether the cirrhosis was of the atrophic type. There can be no doubt that in those cases of hypertrophic cirrhosis the ascites is occasionally spontaneously absorbed, and such cases when operated upon have recovered, notwithstanding the operative interference, and not by reason of it. In some cases it is absolutely impossible to determine the remaining functional value of the diseased liver, and, therefore, impossible to separate the cases that are operable from those that are not. On the whole the author does not believe in this method of treatment. Frazier [8] believes that the operation will prove useful in the following cases:—(1) Those in which the liver is cirrhotic; (2) those in which there is reason to believe the liver-cells are not devoid of function; (3) those in which internal medication (particularly iodide of potassium) and paracentesis fail to afford relief, or, in other words, in utterly hopeless cases; (4) those in which there is no reasonable complication. Moullin[9] thinks that with better selection and earlier operation the mortality should not be appreciably higher than that of exploratory laparotomy. A median incision above the umbilicus is the most convenient, and gives least trouble afterwards. The fluid always collects again after operation, but it collects in the lower part of the abdomen—where it can be drawn off if excessive—and does not interfere with the formation of adhesions. Thompson[10] thinks that the operation is most successful in those cases in which the ascites is caused by peritonitis resulting from the cirrhosis. The cure, when it takes place, is presumably due to obliteration of the peritoneal cavity by the formation of adhesions, just as pleurisy is cured by the adhesion of the layers of the pleura.

**Hydatid Disease of the Gall-Bladder.**—A case of this rare disease is quoted by McGavin.[11] At the time of operation it was supposed to be a case of primary carcinoma of the gall-bladder and cholecystectomy was successfully performed. He has been unable to find more than three cases of hydatid of the gall-bladder recorded in the last twenty years. Apparently none of the cases has been diagnosed before operation and it is doubtful if a definite diagnosis is possible.

[5] Brit. Med. Journ., Jan. 18, 1902, p. 125. [6] Annals of Surgery, July, 1901, p. 166. [7] Amer. Journ. Med. Sci., March, 1902, p. 537. [8] Annals of Surgery, June 1901, p. 715. [9] Brit. Med. Journ., Oct. 19, 1901, p. 1169. [10] Ibid. [11] Lancet, Feb. 22, 1902, p. 504.

